# Emended description of *Actinomyces naeslundii* and descriptions of *Actinomyces oris* sp. nov. and *Actinomyces johnsonii* sp. nov., previously identified as *Actinomyces naeslundii* genospecies 1, 2 and WVA 963

**DOI:** 10.1099/ijs.0.000950-0

**Published:** 2009-03

**Authors:** Uta Henssge, Thuy Do, David R. Radford, Steven C. Gilbert, Douglas Clark, David Beighton

**Affiliations:** 1 Department of Microbiology, The Henry Wellcome Laboratories for Microbiology and Salivary Research, King's College London Dental Institute, Floor 17, Tower Wing, Great Maze Pond, London SE1 9RT, UK; 2 Biomedical Research Centre, Guy's and St Thomas Foundation Trust Hospital, London SE1 9RT, UK

## Abstract

*Actinomyces naeslundii* is an important early colonizer in the oral biofilm and consists of three genospecies (1, 2 and WVA 963) which cannot be readily differentiated using conventional phenotypic testing or on the basis of 16S rRNA gene sequencing. We have investigated a representative collection of type and reference strains and clinical and oral isolates (*n*=115) and determined the partial gene sequences of six housekeeping genes (*atpA*, *rpoB*, *pgi*, *metG*, *gltA* and *gyrA*). These sequences identified the three genospecies and differentiated them from *Actinomyces viscosus* isolated from rodents. The partial sequences of *atpA* and *metG* gave best separation of the three genospecies. *A. naeslundii* genospecies 1 and 2 formed two distinct clusters, well separated from both genospecies WVA 963 and *A. viscosus*. Analysis of the same genes in other oral *Actinomyces* species (*Actinomyces gerencseriae*, *A. israelii*, *A. meyeri*, *A. odontolyticus* and *A. georgiae*) indicated that, when sequence data were obtained, these species each exhibited <90 % similarity with the *A. naeslundii* genospecies. Based on these data, we propose the name *Actinomyces oris* sp. nov. (type strain ATCC 27044^T^ =CCUG 34288^T^) for *A. naeslundii* genospecies 2 and *Actinomyces johnsonii* sp. nov. (type strain ATCC 49338^T^ =CCUG 34287^T^) for *A. naeslundii* genospecies WVA 963. *A. naeslundii* genospecies 1 should remain as *A. naeslundii* *sensu stricto*, with the type strain ATCC 12104^T^ =NCTC 10301^T^ =CCUG 2238^T^.

## INTRODUCTION

*Actinomyces naeslundii* is a major component of the oral biofilm (Li *et al.*, 2004; Marsh & Martin, 1999). Identification of the species is problematic, being thoroughly investigated, but not necessarily resolved, by Johnson *et al.* (1990). Prior to these genetic studies, *A. naeslundii* and *Actinomyces viscosus* from humans were identified using a range of biochemical and physiological tests and were differentiated on the basis of catalase production (Ellen, 1976), with numerous serotypes being recognized amongst these strains (Fillery *et al.*, 1978; Gerencser & Slack, 1976; Putnins & Bowden, 1993). Using DNA–DNA relatedness, Johnson *et al.* (1990) demonstrated that strains identified as *A. naeslundii* serotype I were genetically distinct from other strains identified as *A. naeslundii* or *A. viscosus* and classified these strains as *A. naeslundii* genospecies 1, while other human strains including *A. naeslundii* serotypes NV, II and III and *A. viscosus* serotype II were indistinguishable and were classified as *A. naeslundii* genospecies 2. Strains identified as *Actinomyces* serotype WVA 963 constituted another distinct genospecies, WVA 963, while rodent strains identified as *A. viscosus* serotype I were also genetically distinct. The mean DNA–DNA relatedness between genospecies 1 and genospecies 2 was 37 %, that between genospecies 2 and WVA 963 was 31 % and that between genospecies 1 and WVA 963 was 43 % (derived from Table 2 of Johnson *et al.*, 1990). As there were no conventional microbiological phenotypic tests to distinguish between these genotypes, other than serological tests, no novel species were described, but this study clearly demonstrated that human and animal strains were not related.

*A. naeslundii* genospecies 2 isolates were demonstrated to bind to *N*-acetyl-*β*-d-galactosamine and acidic proline-rich proteins and to exhibit an *N*-acetyl-*β*-d-galactosamine-binding specificity signified by *N*-acetyl-*β*-d-galactosamine-inhibitable coaggregation with specified streptococcal strains. *A. naeslundii* genospecies 1 also bound to *N*-acetyl-*β*-d-galactosamine, but generally not to acidic proline-rich proteins, and possessed another *N*-acetyl-*β*-d-galactosamine-binding specificity to a different set of streptococcal isolates (Hallberg *et al.*, 1998). However, the haemagglutination patterns of strains ascribed to genospecies 1 or 2 were not uniform, indicating phenotypic heterogeneity of the surface properties. It is clear that these phenotypic characteristics are not robust enough to permit the ready or convenient identification of *A. naeslundii* genospecies from oral or clinical samples in disparate laboratories.

Identification of bacteria using 16S rRNA gene sequence comparison is widely used but for some taxa, including viridans streptococci (Hoshino *et al.*, 2005), lactobacilli (Naser *et al.*, 2007) and *Veillonella* species (Jumas-Bilak *et al.*, 2004), this approach is not reliable, and sequence analysis of other genes, including *sodA*, *pheS*, *rpoA*, *rpoB* and *dnaK*, has been used to identify members of these genera. 16S rRNA gene sequence comparison may not be the most reliable method for identifying the *A. naeslundii* genospecies (Tang *et al.*, 2003). Furthermore, strains of genospecies 1 and 2 exhibit >99 % 16S rRNA gene sequence similarity and genospecies WVA 963 strains exhibit >98.5 % similarity with the other two genospecies (see Supplementary Tables S1 and S2, available in IJSEM Online).

We have used partial gene sequence comparison of type and reference strains of *A. naeslundii* genospecies to analyse the relationships between these taxa and propose that *A. naeslundii* genospecies 2 be named *Actinomyces oris* sp. nov. and *A. naeslundii* genospecies WVA 963 be named *Actinomyces johnsonii* sp. nov. and that *A. naeslundii* genospecies 1 remains as *A. naeslundii* *sensu stricto* (Thompson & Lovestedt, 1951); the species can be differentiated by comparison of partial gene sequences of *atpA* or *metG*.

## METHODS

### Bacterial strains.

The type and reference strains of *A. naeslundii* and *A. viscosus* used in this study are shown in Table 1. Identification of isolates was made on the basis of DNA–DNA relatedness analysis (Johnson *et al.*, 1990) or agglutination reactions with genospecies-specific antisera (Putnins & Bowden, 1993) as indicated. We also examined isolates from human extra-oral infections (*n*=12) and 77 isolates from oral samples (plaque and carious dentine) to test the robustness of the proposed method of identification. These isolates were identified as *A. naeslundii*–*A. viscosus* from partial 16S rRNA gene sequences, obtained with universal primer 357F (Lane, 1991), and exhibited >99 % sequence similarity when analysed using blast (http://www.ncbi.nlm.nih.gov/blast/Blast.cgi). The clinical and oral isolates are listed in Supplementary Table S3. All isolates were subcultured on fastidious anaerobe agar (FAA; LabM Ltd), grown anaerobically overnight at 37 °C and preserved in glycerol-containing medium at −80 °C. *Actinomyces gerencseriae* ATCC 23860^T^, *Actinomyces israelii* ATCC 12102^T^, *Actinomyces meyeri* ATCC 35568^T^, *Actinomyces odontolyticus* NCTC 9935^T^ and *Actinomyces georgiae* R11726 were included in the sequence analyses for comparative purposes.

### Biochemical tests.

All isolates were tested using the API Rapid ID32A kit (bioMérieux) according to the manufacturer's instructions and were tested for aesculin hydrolysis and for acid production from arabinose, cellobiose, fructose, glycogen, inositol, lactose, mannitol, ribose and trehalose (at 1 % w/v) and salicin and starch (at 0.5 % w/v) in peptone-yeast extract broth as described previously (Brailsford *et al.*, 1999). Isolates were also tested for the presence of preformed glycosidic enzyme activities (*N*-acetyl-*β*-glucosaminidase, *N*-acetyl-*β*-galactosaminidase, *α*-l-fucosidase, *β*-d-fucosidase, sialidase, *β*-glucosidase, *α*-glucosidase, *α*-arabinosidase, *α*-galactosidase and *β*-galactosidase) with 4-methylumbelliferyl-linked fluorogenic substrates as described previously (Beighton *et al.*, 1991). The catalase activity and ability of each isolate to grow in air was also determined.

### Housekeeping genes.

Six housekeeping genes [*atpA* (ATP synthase F1, alpha subunit, ANA_0169), *rpoB* (DNA-directed RNA polymerase, beta subunit, ANA_1497), *pgi* (glucose-6-phosphate isomerase, ANA_0727), *metG* (methionyl-tRNA synthase, ANA_1898), *gltA* (citrate synthase I, ANA_1674) and *gyrA* (DNA gyrase, subunit A, ANA_2224)] were identified from the genome of *A. naeslundii* MG1 (http://cmr.jcvi.org/tigr-scripts/CMR/CmrHomePage.cgi). These genes were selected as they were present as single copies in the MG1 genome, were widely spaced on the chromosome and were of sufficient size for primer design to yield amplicons of >450 bp. The primers used in the primary amplifications and for sequencing and amplicon sizes are shown in Table 2.

### Gene amplification and DNA sequencing.

To extract DNA from isolates, they were grown overnight on FAA and bacteria were washed in 2 M NaCl. Cells were resuspended in TE buffer containing 0.5 % Tween 20 (pH 8.0) and proteinase K was added to a final concentration of 200 μg ml^−1^ (Aas *et al.*, 2005). The tubes were incubated at 55 °C for 2 h and subsequently heated at 95 °C for 5 min to inactivate the proteinase K. DNA extracts were stored at −20 °C.

PCRs to amplify the individual genes were performed in a total volume of 15 μl composed of 1 μl DNA template, 0.2 μM each primer, 2 mM MgCl_2_ and Reddy-Mix (Thermo Scientific). Because of noticeable sequence variability and/or recurring high-G+C regions, each gene was amplified simultaneously using multiple PCR primer sets (Table 2). After heating, DNA was amplified with 30 cycles and an annealing temperature of 53 °C. Prior to sequencing, the PCR products were purified by adding 4 U exonuclease I (Fermentas) and 1 U shrimp alkaline phosphatase (Thermo Scientific) to each reaction and incubating at 37 °C for 45 min; the enzymes were then inactivated at 80 °C for 15 min. Amplicon sequencing of both strands was performed by using the ABI Prism BigDye Terminator Sequencing kit (Applied Biosystems) with 30 cycles of denaturation at 96 °C for 10 s, annealing at 50 °C for 5 s and extension at 60 °C for 2 min. Sequencing reaction products were run on an ABI sequencer 3730xl (Applied Biosystems).

### Sequence analysis.

All DNA sequences were analysed, trimmed and aligned using BioEdit software (version 7.0.0; http://www.mbio.ncsu.edu/BioEdit/bioedit.html). Phylogenetic relationships between the type and reference strains and the human oral and clinical isolates were analysed using mega 3.1 (Kumar *et al.*, 2004). Distances were calculated using Kimura's two-parameter model and, for clustering, the neighbour-joining method of Saitou & Nei (1987) was employed using bootstrap values based on 500 replicates.

## RESULTS AND DISCUSSION

### Differentiation of genospecies by sequence analysis

**Fig. 1. f1:**
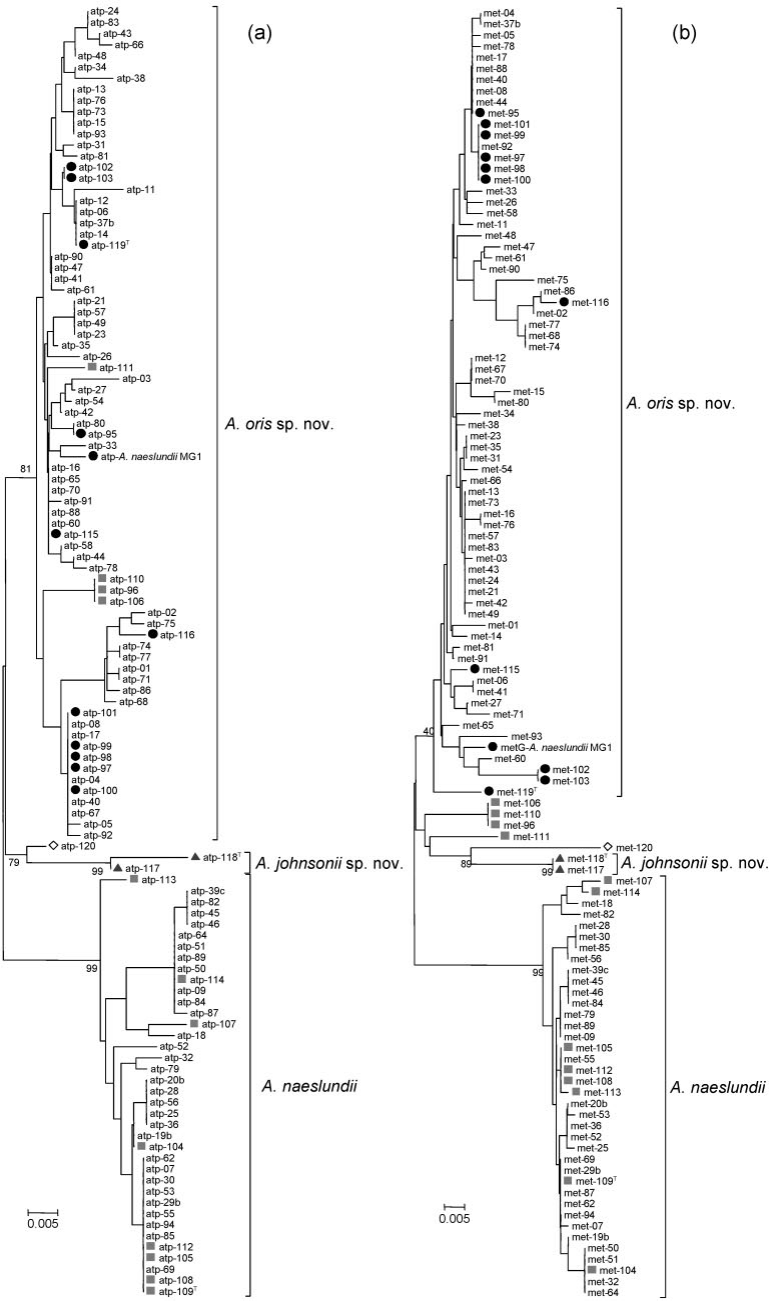
Neighbour-joining tree showing taxonomic relationships between type and reference strains of *A. naeslundii*, *A. oris* sp. nov., *A. johnsonii* sp. nov. and *A. viscosus* and oral and clinical isolates determined by partial gene sequence analysis of *atpA* (a) and *metG* (b). Type and reference strains are identified by study numbers (see Table 1) and indicated as follows: •, *A. oris* sp. nov. (genospecies 2); ▪, *A. naeslundii* (genospecies 1); ◊, *A. viscosus*; ▴, *A. johnsonii* sp. nov. (genospecies WVA 963). Bootstrap values are indicated at corresponding nodes. See Supplementary Fig. S1 for dendrograms for *rpoB*, *gyrA*, *pgi* and *gltA*. Bars, 0.005 substitutions per site.

The sequence heterogeneity within each gene required the use of sets of primers for each targeted gene to produce amplicons for use in nested PCRs. The pairs of sequencing primers successfully sequenced each of the gene fragments from the majority of organisms but, in a small number of cases, one of the initial amplification primers was required. Neighbour-joining trees for each of the six housekeeping genes (*atpA*, *metG*, *rpoB*, *gyrA*, *pgi* and *gltA*) are shown in Fig. 1 and in Supplementary Fig. S1. There was very good agreement between the genotype of the isolate as received and the cluster with which each strain was associated on the basis of the partial gene sequences. The only difficulty was found with strains P6N, P10N, P5N and P11N (=CCUG 33920), which were received as genospecies 1, but which were found to align with the type and reference strains designated genospecies 2 with each of the six housekeeping genes. The initial assignment of these four strains to genospecies 1 was made on the basis of their agglutination reactions with genospecies-specific antisera, but the haemagglutination reactions of these strains were distinct from others designated genospecies 1, as they failed to haemagglutinate intact human, goat, sheep or horse red blood cells while the other strains designated genotype 1 haemagglutinated these cells (Hallberg *et al.*, 1998). The present data suggest that genospecies-specific antisera (Putnins & Bowden, 1993) may not always be reliable in identifying genospecies 2 isolates, as some may be misidentified as genospecies 1, and reassessment of previous data might be necessary. It also follows that there is greater homogeneity within the haemagglutination reactions of genospecies 1 than was previously recognized.

The dendrograms had similar overall topographies but differed with respect to the distance between genospecies 1 and genospecies 2 clusters and the sequence heterogeneity within the genospecies clusters. The finding that the tree topologies are not identical does not limit their use in assigning isolates to a particular species, since various factors may account for the individual tree topologies, including the level of information content, different rates of evolution due to selective pressures and the length of the partial sequences that are compared (Christensen *et al.*, 2004). The variation in the discriminatory power of individual genes suggests the use of multiple genes for the most robust identification of isolates (Naser *et al.*, 2007).

In all dendrograms, the two genospecies WVA 963 strains were distinct from genospecies 1 and 2 strains, confirming the conclusions from DNA–DNA relatedness data that these strains are genetically distinct (Johnson *et al.*, 1990) and refuting the suggestion that isolates identified as serotype WVA 963 should be included in genospecies 2 (Putnins & Bowden, 1993). The single rodent *A. viscosus* serotype I strain (the type strain) was also distinct from the *A. naeslundii* strains.

The different housekeeping genes were not equally able to separate the genospecies. The sequences of *atpA* were the most homogeneous for genospecies 2, and all genospecies were well separated. The *metG* gene also exhibited the greatest sequence difference between genospecies 1 and 2; each genospecies was characterized by low heterogeneity within the cluster and the hamster strain and genotype WVA 963 strains were readily differentiated. The *rpoB* gene was less heterogeneous in genospecies 1 than in genospecies 2 but both clusters, and genospecies WVA 963 and the hamster strain, were well separated. With *gyrA*, genospecies 1 and 2 could be readily differentiated but, within each genospecies, there was considerable sequence diversity. The sequence variations for *pgi* within the genospecies and the hamster strain of *A. viscosus* may not be sufficient to permit reliable identification of the isolates. The phylogenetic tree of *gltA* indicated greater homogeneity within strains of genospecies 1 but also greater heterogeneity within genospecies 2, which were not as well separated from genospecies 1 strains, or from *A. viscosus* or genospecies WVA963, as they were with *atpA* and *metG*. Overall, the genes *aptA*, *metG*, *rpoB* and *gyrA* exhibited the most discrete clusters for genospecies 1 and 2. In each dendrogram, the two strains of genospecies WVA 963 were positioned between the other two genospecies and always occurred together. The animal strain *A. viscosus* NCTC 10951^T^ branched either together with the strains of genospecies WVA 963 with *atpA*, *gyrA*, *metG* and *pgi* or separately between genospecies 1 and 2 with *gltA* and *rpoB*.

The partial sequences of all six housekeeping genes differed markedly in the other five oral *Actinomyces* species investigated. PCR products or sequence data from both strands could not be obtained for all strains. For *atpA*, sequence data were obtained only for *A. meyeri* ATCC 35568^T^, *A. georgiae* R11726 and *A. gerencseriae* ATCC 23860^T^, but the sequence similarity was <91 % with the *A. naeslundii* genotype strains. *metG* sequences were obtained for *A*. *israelii* ATCC 12102^T^ and *A*. *odontolyticus* NCTC 9935^T^, and they had <86 % similarity with the *A. naeslundii* strains, while an *rpoB* sequence was obtained only for *A*. *israelii* ATCC 12102^T^, which had <90 % similarity. The gene *gyrA* could not be sequenced in *A*. *israelii* ATCC 12102^T^ or *A*. *georgiae* R11726, and the other strains had <87 % sequence similarity with the *A. naeslundii* genotype strains and, with *pgi*, no sequence could be obtained for *A*. *israelii* ATCC 12102^T^ and the similarity between the species and the *A. naeslundii* genotypes was <92 %. Sequences for *gltA* were only obtained from *A*. *israelii* ATCC 12102^T^ and *A*. *gerencseriae* ATCC 23860^T^, and they exhibited <88 % similarity with the *A. naeslundii* genotype strains.

On the basis of discrimination between the 12 reference and type strains, all of the sequences from the oral and clinical isolates could be assigned to either *A. naeslundii* genospecies 1 or 2. The non-oral clinical isolates (study numbers 83–94) and the oral clinical isolates (study numbers 1–82) were each detected in the same cluster with each of the genes. Of the 89 oral and non-oral clinical isolates, 59 were identified as genospecies 2 and 30 were identified as genospecies 1.

### Phenotypic characterization of the resulting clusters

For phenotypic description, all isolates were tested with the API Rapid ID32A kit, and additional carbohydrate fermentations and enzyme reactions were carried out. None of the 115 isolates were identified as *A. naeslundii* or *A. viscosus* at an acceptable level using the API Rapid ID32A kit. The percentage of positive reactions for each test for the three *A. naeslundii* genospecies, as assigned from the phylogenetic analysis, is listed in Table 3. No distinctive patterns enabled the use of these tests to distinguish between the genospecies, confirming the extensive phenotypic data reported previously (Johnson *et al.*, 1990).

### Description of *Actinomyces oris* sp. nov.

*Actinomyces oris* (o′ris. L. gen. n. *oris* of the mouth).

Contains strains previously identified as *A. naeslundii* serotypes II, III and NV and *A. viscosus* serotype II. Formerly known as *Actinomyces naeslundii* genospecies 2 and, on the basis of conventional phenotypic testing, is indistinguishable from other *A. naeslundii* genotypes. Biochemical and physiological characteristics are as reported for *A. naeslundii* genospecies 2 (Johnson *et al.*, 1990) supplemented with those reported here. The G+C content of the type strain is 66 mol%. *Actinomyces oris* may be differentiated from closely related species on the basis of sequence comparisons of partial gene sequences of *atpA* or *metG*.

The type strain is ATCC 27044^T^ (=CCUG 34288^T^), isolated from human sputum.

### Description of *Actinomyces johnsonii* sp. nov.

*Actinomyces johnsonii* (john.so′ni.i. N.L. gen. n. *johnsonii* of Johnson, named after the American molecular taxonomist John L. Johnson, who undertook extensive studies on the genetic relationships between oral actinomyces).

Contains strains previously identified as *A. naeslundii* serotype WVA 963. Formerly known as *Actinomyces naeslundii* genospecies WVA 963 and, on the basis of conventional phenotypic testing, is indistinguishable from other *A. naeslundii* genotypes. Biochemical and physiological characteristics are as reported for *A. naeslundii* genospecies WVA 963 (Johnson *et al.*, 1990). The G+C content of the type strain is 67 mol%. *Actinomyces johnsonii* may be differentiated from closely related species on the basis of sequence comparisons of partial gene sequences of *atpA* or *metG*.

The type strain is ATCC 49338^T^ (=CCUG 34287^T^), isolated from the gingival crevice of a healthy child.

### Emended description of *Actinomyces naeslundii* Thompson and Lovestedt 1951

Contains strains previously identified as *A. naeslundii* serotype I. Formerly known as *Actinomyces naeslundii* genospecies 1 and, on the basis of conventional phenotypic testing, is indistinguishable from other *A. naeslundii* genotypes. Biochemical and physiological characteristics are as reported for *A. naeslundii* genospecies 1 (Johnson *et al.*, 1990) supplemented with those reported here. The G+C content of the type strain is 66 mol%. *Actinomyces naeslundii* may be differentiated from closely related species on the basis of sequence comparisons of partial gene sequences of *atpA* or *metG*.

The type strain is ATCC 12104^T^ =NCTC 10301^T^ =CCUG 2238^T^, isolated from a human sinus.

## Supplementary Material

[Supplementary Tables and Figure]

## Figures and Tables

**Table 1. t1:** Type and reference strains used in this study Strains labelled Strömberg were kindly provided by Professor Nicolas Strömberg, University of Göteborg, Sweden; strains labelled CCUG were purchased directly from the Culture Collection of the University of Göteborg. *A. viscosus* NCTC 10951^T^ was purchased from the National Collection of Type Cultures, HPA, Colindale, UK.

**Strain**	**Source**	**Clinical origin**	**Study no.**
**Received as genospecies 1**			
P6N	Strömberg	Plaque	96
Pn1GA	Strömberg	Plaque	104
Pn16E	Strömberg	Plaque	105
P10N	Strömberg	Plaque	106
Pn6N	Strömberg	Plaque	107
Pn20E	Strömberg	Plaque	108
ATCC 12104^T^	Strömberg	Plaque	109
P5N	Strömberg	Plaque	110
P11N (=CCUG 33920)	CCUG	Plaque	111
461 (=CCUG 34725)	CCUG	Occlusal plaque	112
TF 11 (=CCUG 35334)	CCUG	Blood (endocarditis)	113
R709-03041/97 (=CCUG 37599)	CCUG	Cerebrospinal fluid	114
**Received as genospecies 2**			
P2G	Strömberg	Plaque	95
P5K	Strömberg	Plaque	97
P6K	Strömberg	Plaque	98
P7K	Strömberg	Plaque	99
P8K	Strömberg	Plaque	100
P9K	Strömberg	Plaque	101
Pn4D	Strömberg	Plaque	102
Pn5D	Strömberg	Plaque	103
VPI 12593 (=CCUG 34285)*	CCUG	Human abscess	115
VPI D163E-3 (=CCUG 34286)*	CCUG	Gingival crevice (periodontitis)	116
ATCC 27044 (*A. viscosus* serotype II)*	ATCC	Human sputum	119
**Received as genospecies WVA 963**			
PK1259 (=CCUG 33932)	CCUG	Subgingival plaque	117
WVA 963 (=CCUG 34287)*	CCUG	Subgingival plaque	118
**Received as *A. viscosus***			
*A. viscosus* serotype I NCTC 10951^T^*	NCTC	Hamster	120

*Genotype determined by DNA–DNA relatedness (Johnson *et al.*, 1990); others were determined by using genospecies-specific antisera (Putnins & Bowden, 1993).

**Table 2. t2:** Primers used to amplify and sequence fragments of housekeeping genes investigated for their ability to identify members of *A. naeslundii* genospecies 1, 2 and WVA 963

**Primer**	**Primer sequence sets (5′–3′)**	**Sequencing primer (5′–3′)**	**Fragment length (bp)**
***atpA***			
AtpA-F	TCGCCGAGTCCTACAAGCACACCATCCTCAACCAGAAACCCGAACAAGCAGGTG	CCCTGGAGTACACCACCAT	474
AtpA-R	CAGGTCGGAAGCGAACATCGGGTAGGGCGTGTACTTCCTCGGTCTCGTCAGTGA	CGCCAGGGTGATCTTGAG	
***gltA***			
GltA-F	GCCAAGTCCTCGACCTTCCGCCTACCTCCTCATCAA	CCAAGATGCCCACGATGAT	552
GltA-R	CGAACAGGGGTGTGAACATGGTGGAGCCGTCGTAAAC	GGCGGTCTCCTTGACGAT	
***gyrA***			
GyrA-F	GCGACGAAGTTGAAGAGATGCCTCCTTCTCCAAGTCCT	GGTACACCGAGTGCAAGAT	564
GyrA-R	GCCGTAGTTGGTGAAGAACGCTCGTCGCCGTACTTCT	CGACCAGGGCGAGCATATT	
***metG***			
MetG-F	GCCGACCGCAACAACGCCTGTCCTACGACCTGTTCACTTCATGGGCAAGGACAA	GAGGTCGTCTCCAGCG	504
MetG-R	CGGAGTCGGCGTAACGACTCGTCCAGCTTGGTGAACGGTGATGATCGGGTAGC	CAGGAAGGGCGAGAGCAT	
***pgi***			
Pgi-F	GAGCTGGGAGACCTCTACATCGACCTGTCCAAGAACCT	GCGAGCACATCAACATCA	531
Pgi-R	TGGTGGATGAGCTGGTAGAGGAAGTCGGCGAAGTTCT	CACCCACCCAGTTCCAGAA	
***rpoB***			
RpoB-F	CGAGGAGCCGAAGTACACGTCACCGTCTTTCTGCCTGGGCATGGACGAGT	GGACGAGGACCGCAAGAT	491
RpoB-R	GGCGTCCTCGTAGTTGACGTAGTGCGAGGTGTGGAGGTTGTTCTGGTCCATGA	TGGGAGGTGCCGAAGAAC	

**Table 3. t3:** Biochemical properties of *A. naeslundii* genospecies 1, 2 and WVA 963 Data are percentages of strains giving positive reactions. Isolates were assigned to individual genospecies on the basis of their positions in phylogenetic trees calculated using partial gene sequences of *atpA* and *metG*. All strains were positive for activity of proline, phenylalanine and leucine arylamidases in the API Rapid ID32A kit and for neuraminidase (sialidase) and *α*-glucosidase activity using fluorogenic substrates, and all exhibited aerobic growth. All strains were negative for arginine dihydrolase, *β*-galactosidase-6-phosphate, *α*-arabinosidase, *β*-glucuronidase, *N*-acetyl-*β*-glucosaminidase, glutamic acid decarboxylase, *α*-fucosidase and for activity of arginine, leucylglycine, pyroglutamic acid, histidine, glutamylglutamic acid and serine arylamidases in the API Rapid ID32A kit and for *N*-acetyl-*β*-d-galactosaminidase and *N*-acetyl-*β*-d-glucosaminidase activities using fluorogenic substrates, none fermented arabinose or mannitol using the previously described method (Brailsford *et al.*, 1999) and none produced indole.

**Phenotypic test**	**Genospecies 1 (*A. naeslundii*) (*n*=42)**	**Genospecies 2 (*A. oris* sp. nov.) (*n*=70)**	**Genospecies WVA 963 (*A. johnsonii* sp. nov.) (*n*=2)**
**API Rapid ID32A**			
Urease	0	1	50
*α*-Galactosidase	44	44	100
*β*-Galactosidase	75	67	100
*α*-Glucosidase	88	81	100
*β*-Glucosidase	83	61	100
Mannose fermentation	0	1	50
Raffinose fermentation	2	4	0
Nitrate reduction	98	96	100
Alkaline phosphatase	0	0	50
Tyrosine arylamidase	98	97	100
Alanine arylamidase	0	1	0
Glycine arylamidase	0	3	0
**Other methods**			
Fermentation of:			
Cellobiose	31	9	0
Fructose	98	99	50
Glycogen	5	40	100
Inositol	100	93	50
Lactose	71	67	100
Ribose	0	21	100
Salicin	64	23	0
Starch	2	16	50
Trehalose	50	43	50
Aesculin hydrolysis	62	13	0
Catalase production	10	70	50
*β*-d-Fucosidase	90	91	100
*α*-l-Fucosidase	12	0	100
*β*-Glucosidase	100	90	100
*α*-Galactosidase	100	96	100
*α*-Arabinosidase	88	93	100
*β*-Galactosidase	88	93	100
