# Supplementary material for: Emended description of Actinomyces naeslundii and descriptions of Actinomyces oris sp. nov. and Actinomyces johnsonii sp. nov., previously identified as Actinomyces naeslundii genospecies 1, 2 and WVA 963
Source: Int J Syst Evol Microbiol. 2009 Mar;59(Pt 3):509–16. doi: 10.1099/ijs.0.000950-0 (PMC2884933; doi:10.1099/ijs.0.000950-0)
Supplement: [Supplementary Tables and Figure] [file supp_59_3_509__index.html]

 Emended description of Actinomyces naeslundii and descriptions of Actinomyces oris sp. nov. and Actinomyces johnsonii sp. nov., previously identified as Actinomyces naeslundii genospecies 1, 2 and WVA 963 -- Henssge et al. 59 (3): 509 Data Supplement - Supplementary Tables and Figure -- International Journal of Systematic and Evolutionary Microbiology 

### Emended description of *Actinomyces naeslundii* and descriptions of *Actinomyces oris* sp. nov. and *Actinomyces johnsonii* sp. nov., previously identified as *Actinomyces naeslundii* genospecies 1, 2 and WVA 963, by U. Henssge, T. Do, D. R. Radford, S. C. Gilbert, D. Clark and D. Beighton

*International Journal of Systematic and Evolutionary Microbiology* vol. **59**, part 3, pp. 509 - 516

**Supplementary Table S1.** 16S rRNA gene sequence similarity between groups

**Supplementary Table S2.** Strains used and accession numbers for partial 16S rRNA gene sequences

**Supplementary Table S3.** Details of oral and non-oral isolates and reference and type strains used in this study and GenBank accession numbers of partial sequences

**Supplementary Fig. S1.** Phylogenetic relationships between *A. naeslundii* genospecies 1 (*A. naeslundii* *sensu stricto*), genospecies 2 (*A. oris* sp. nov.) and serotype WVA 963 (*A. johnsonii* sp. nov.), generated by neighbour-joining analysis of partial gene sequences of *rpoB*, *gyrA*, *pgi* and *gltA*

[PDF file of Supplementary Tables and Figure] (92 KB)

  
  
