# Supplementary material for: Emended description of Actinomyces naeslundii and descriptions of Actinomyces oris sp. nov. and Actinomyces johnsonii sp. nov., previously identified as Actinomyces naeslundii genospecies 1, 2 and WVA 963
Source: Int J Syst Evol Microbiol. 2009 Mar;59(Pt 3):509–16. doi: 10.1099/ijs.0.000950-0 (PMC2884933; doi:10.1099/ijs.0.000950-0)
Supplement: [Supplementary Tables and Figure] [file supp_59_3_509__1.pdf]

**Supplementary Table S1.** 16S rRNA gene sequence similarity between groups

Values are mean percentages calculated using Kimura's two-parameter correction.

| **Group** | ***A. oris* sp. nov. Genospecies 2** | ***A. naeslundii* Genospecies 1** | ***A. johnsonii* sp. nov. Serotype WVA 963** |
|---|---|---|---|
| *A. naeslundii* | 99.2 | – | |
| *A. johnsonii* sp. nov. | 98.7 | 98.9 | – |
| *A. viscosus* | 99.2 | 99.2 | 98.5 |

**Supplementary Table S2.** Strains used and accession numbers for partial 16S rRNA gene sequences

| **Strain** | **Accession number** | **Species name** | **Source** | **Study no.** |
|---|---|---|---|---|
| P2G | EU667389 | *A. oris* | Strömberg | 95 |
| P6N | EU667390 | *A. oris* | Strömberg | 96 |
| P5K | EU667391 | *A. oris* | Strömberg | 97 |
| P6K | EU667392 | *A. oris* | Strömberg | 98 |
| P7K | EU667393 | *A. oris* | Strömberg | 99 |
| P8K | EU667394 | *A. oris* | Strömberg | 100 |
| P9K | EU667395 | *A. oris* | Strömberg | 101 |
| Pn4D | EU667396 | *A. oris* | Strömberg | 102 |
| Pn5D | EU667397 | *A. oris* | Strömberg | 103 |
| Pn1GA | EU667398 | *A. naeslundii* | Strömberg | 104 |
| Pn16E | EU667399 | *A. naeslundii* | Strömberg | 105 |
| P10N | EU667400 | *A. oris* | Strömberg | 106 |
| Pn20E | EU667401 | *A. naeslundii* | Strömberg | 108 |
| P5N | EU667402 | *A. oris* | Strömberg | 110 |
| [*A. viscosus*] ATCC 27044$^T$ | EU667403 | *A. oris* | ATCC | 119 |
| P11N (=CCUG 33920) | EU667404 | *A. oris* | CCUG | 111 |
| PK1259 (=CCUG 33932) | EU667405 | *A. johnsonii* | CCUG | 117 |
| VPI 12593 (=CCUG 34285) | EU667406 | *A. oris* | CCUG | 115 |
| VPI D163E-3 (=CCUG 34286) | EU667407 | *A. oris* | CCUG | 116 |
| 461 (=CCUG 34725) | EU667408 | *A. naeslundii* | CCUG | 112 |
| TF 11 (=CCUG 35334) | EU667409 | *A. naeslundii* | CCUG | 113 |
| R709-03041/97 (=CCUG 37599) | EU667410 | *A. naeslundii* | CCUG | 114 |
| CCUG 34287$^T$ | EU667411 | *A. johnsonii* | CCUG | 118 |

**Supplementary Table S3.** Details of oral and non-oral isolates and reference and type strains used in this study and GenBank accession numbers of partial sequences

| Study no. | Isolate | Origin | GenBank accession no. | | | | | |
|---|---|---|---|---|---|---|---|---|
| | | | *atpA* | *gltA* | *gyrA* | *metG* | *pgi* | *rpoB* |
| **Oral and non-oral clinical isolates** | | | | | | | | |
| 01 | A18A-3 | Leathery lesion | EU620779 | EU620895 | EU621011 | EU621127 | EU603149 | EU621243 |
| 02 | A19A-1 | Leathery lesion | EU620780 | EU620896 | EU621012 | EU621128 | EU603150 | EU621244 |
| 03 | A2A-1 | Leathery lesion | EU620781 | EU620897 | EU621013 | EU621129 | EU603151 | EU621245 |
| 04 | A3A-1 | Leathery lesion | EU620782 | EU620898 | EU621014 | EU621130 | EU603152 | EU621246 |
| 05 | A6A-1 | Leathery lesion | EU620783 | EU620899 | EU621015 | EU621131 | EU603153 | EU621247 |
| 06 | A7A-1 | Leathery lesion | EU620784 | EU620900 | EU621016 | EU621132 | EU603154 | EU621248 |
| 07 | E1-20 | Plaque (caries –) | EU620785 | EU620901 | EU621017 | EU621133 | EU603155 | EU621249 |
| 08 | F11C1 | Plaque (caries +) | EU620786 | EU620902 | EU621018 | EU621134 | EU603156 | EU621250 |
| 09 | F12B1 | Plaque (caries +) | EU620787 | EU620903 | EU621019 | EU621135 | EU603157 | EU621251 |
| 11 | F1A1 | Plaque (caries –) | EU620788 | EU620904 | EU621020 | EU621136 | EU603158 | EU621252 |
| 12 | F21A1 | Plaque (caries +) | EU620789 | EU620905 | EU621021 | EU621137 | EU603159 | EU621253 |
| 13 | F23A1 | Plaque (caries +) | EU620790 | EU620906 | EU621022 | EU621138 | EU603160 | EU621254 |
| 14 | F24C1 | Plaque (caries +) | EU620791 | EU620907 | EU621023 | EU621139 | EU603161 | EU621255 |
| 15 | F2A12 | Plaque (caries –) | EU620792 | EU620908 | EU621024 | EU621140 | EU603162 | EU621256 |
| 16 | F4D1 | Plaque (caries –) | EU620793 | EU620909 | EU621025 | EU621141 | EU603163 | EU621257 |
| 17 | F5A10 | Plaque (caries –) | EU620794 | EU620910 | EU621026 | EU621142 | EU603164 | EU621258 |
| 18 | F6E1 | Plaque (caries –) | EU620795 | EU620911 | EU621027 | EU621143 | EU603165 | EU621259 |
| 19b | G126D | Plaque (caries –) | EU620796 | EU620912 | EU621028 | EU621144 | EU603166 | EU621260 |
| 20b | G127B | Plaque (caries –) | EU620797 | EU620913 | EU621029 | EU621145 | EU603167 | EU621261 |
| 21 | G128C | Plaque (caries –) | EU620798 | EU620914 | EU621030 | EU621146 | EU603168 | EU621262 |
| 23 | G140C | Plaque (caries –) | EU620799 | EU620915 | EU621031 | EU621147 | EU603169 | EU621263 |
| 24 | G48B | Plaque (caries –) | EU620800 | EU620916 | EU621032 | EU621148 | EU603170 | EU621264 |
| 25 | G51C | Plaque (caries –) | EU620801 | EU620917 | EU621033 | EU621149 | EU603171 | EU621265 |
| 26 | G53E | Plaque (caries –) | EU620802 | EU620918 | EU621034 | EU621150 | EU603172 | EU621266 |
| 27 | G54C | Plaque (caries –) | EU620803 | EU620919 | EU621035 | EU621151 | EU603173 | EU621267 |
| 28 | T14P-1 | Plaque (caries –) | EU620804 | EU620920 | EU621036 | EU621152 | EU603174 | EU621268 |
| 29b | T17-3 | Plaque (caries –) | EU620805 | EU620921 | EU621037 | EU621153 | EU603175 | EU621269 |
| 30 | T20P-1 | Plaque (caries –) | EU620806 | EU620922 | EU621038 | EU621154 | EU603176 | EU621270 |
| 31 | T22P-A1 | Plaque (caries –) | EU620807 | EU620923 | EU621039 | EU621155 | EU603177 | EU621271 |
| 32 | T23P-1 | Plaque (caries –) | EU620808 | EU620924 | EU621040 | EU621156 | EU603178 | EU621272 |
| 33 | T5P-1 | Plaque (caries –) | EU620809 | EU620925 | EU621041 | EU621157 | EU603179 | EU621273 |
| 34 | T6P-2 | Plaque (caries –) | EU620810 | EU620926 | EU621042 | EU621158 | EU603180 | EU621274 |
| 35 | T8-1 | Plaque (caries –) | EU620811 | EU620927 | EU621043 | EU621159 | EU603181 | EU621275 |
| 36 | T9P-1 | Plaque (caries –) | EU620812 | EU620928 | EU621044 | EU621160 | EU603182 | EU621276 |
| 37b | UN4B6c | Plaque (caries +) | EU620813 | EU620929 | EU621045 | EU621161 | EU603183 | EU621277 |
| 38 | W11-1-1 | Plaque (caries +) | EU620814 | EU620930 | EU621046 | EU621162 | EU603184 | EU621278 |
| 39c | W8-2-3 | Plaque (caries +) | EU620815 | EU620931 | EU621047 | EU621163 | EU603185 | EU621279 |
| 40 | WE10B-1 | Plaque (caries +) | EU620816 | EU620932 | EU621048 | EU621164 | EU603186 | EU621280 |
| 41 | WE1Aa1 | Plaque (caries +) | EU620817 | EU620933 | EU621049 | EU621165 | EU603187 | EU621281 |

| Study no. | Isolate | Origin | GenBank accession no. | | | | | |
|---|---|---|---|---|---|---|---|---|
| | | | ***atpA*** | ***gltA*** | ***gyrA*** | ***metG*** | ***pgi*** | ***rpoB*** |
| 42 | WE3Bc14 | Plaque (caries +) | EU620818 | EU620934 | EU621050 | EU621166 | EU603188 | EU621282 |
| 43 | WE4Bb1 | Plaque (caries +) | EU620819 | EU620935 | EU621051 | EU621167 | EU603189 | EU621283 |
| 44 | WE5Ba2 | Plaque (caries +) | EU620820 | EU620936 | EU621052 | EU621168 | EU603190 | EU621284 |
| 45 | WE6B-3 | Plaque (caries +) | EU620821 | EU620937 | EU621053 | EU621169 | EU603191 | EU621285 |
| 46 | WE7B1 | Plaque (caries +) | EU620822 | EU620938 | EU621054 | EU621170 | EU603192 | EU621286 |
| 47 | S24V | Plaque (caries –) | EU620823 | EU620939 | EU621055 | EU621171 | EU603193 | EU621287 |
| 48 | S29C | Plaque (caries –) | EU620824 | EU620940 | EU621056 | EU621172 | EU603194 | EU621288 |
| 49 | S33A | Plaque (caries –) | EU620825 | EU620941 | EU621057 | EU621173 | EU603195 | EU621289 |
| 50 | S38H | Plaque (caries –) | EU620826 | EU620942 | EU621058 | EU621174 | EU603196 | EU621290 |
| 51 | S41C | Plaque (caries –) | EU620827 | EU620943 | EU621059 | EU621175 | EU603197 | EU621291 |
| 52 | S43L | Plaque (caries –) | EU620828 | EU620944 | EU621060 | EU621176 | EU603198 | EU621292 |
| 53 | S44D | Plaque (caries –) | EU620829 | EU620945 | EU621061 | EU621177 | EU603199 | EU621293 |
| 54 | S47B | Plaque (caries –) | EU620830 | EU620946 | EU621062 | EU621178 | EU603200 | EU621294 |
| 55 | S49H | Plaque (caries –) | EU620831 | EU620947 | EU621063 | EU621179 | EU603201 | EU621295 |
| 56 | S53C | Plaque (caries –) | EU620832 | EU620948 | EU621064 | EU621180 | EU603202 | EU621296 |
| 57 | S55N | Plaque (caries –) | EU620833 | EU620949 | EU621065 | EU621181 | EU603203 | EU621297 |
| 58 | S56D | Plaque (caries –) | EU620834 | EU620950 | EU621066 | EU621182 | EU603204 | EU621298 |
| 60 | S62B | Plaque (caries –) | EU620835 | EU620951 | EU621067 | EU621183 | EU603205 | EU621299 |
| 61 | S64C | Plaque (caries –) | EU620836 | EU620952 | EU621068 | EU621184 | EU603206 | EU621300 |
| 62 | S65A | Plaque (caries –) | EU620837 | EU620953 | EU621069 | EU621185 | EU603207 | EU621301 |
| 64 | U136-1 | Leathery lesion | EU620838 | EU620954 | EU621070 | EU621186 | EU603208 | EU621302 |
| 65 | U149-1 | Leathery lesion | EU620839 | EU620955 | EU621071 | EU621187 | EU603209 | EU621303 |
| 66 | MMRCO1-1 | Soft lesion | EU620840 | EU620956 | EU621072 | EU621188 | EU603210 | EU621304 |
| 67 | MMRCO2-1 | Soft lesion | EU620841 | EU620957 | EU621073 | EU621189 | EU603211 | EU621305 |
| 68 | MMRCO6-1 | Soft lesion | EU620842 | EU620958 | EU621074 | EU621190 | EU603212 | EU621306 |
| 69 | M42-1-1 | Plaque (caries +) | EU620843 | EU620959 | EU621075 | EU621191 | EU603213 | EU621307 |
| 70 | M46-1-1 | Plaque (caries +) | EU620844 | EU620960 | EU621076 | EU621192 | EU603214 | EU621308 |
| 71 | M47-1-1 | Plaque (caries +) | EU620845 | EU620961 | EU621077 | EU621193 | EU603215 | EU621309 |
| 73 | F7j1 | Plaque (caries –) | EU620846 | EU620962 | EU621078 | EU621194 | EU603216 | EU621310 |
| 74 | F28B1 | Plaque (caries +) | EU620847 | EU620963 | EU621079 | EU621195 | EU603217 | EU621311 |
| 75 | WE8B-23 | Plaque (caries +) | EU620848 | EU620964 | EU621080 | EU621196 | EU603218 | EU621312 |
| 76 | WE9A10-1 | Plaque (caries +) | EU620849 | EU620965 | EU621081 | EU621197 | EU603219 | EU621313 |
| 77 | M48-1B-1 | Plaque (caries +) | EU620850 | EU620966 | EU621082 | EU621198 | EU603220 | EU621314 |
| 78 | M67-1 | Plaque (caries +) | EU620851 | EU620967 | EU621083 | EU621199 | EU603221 | EU621315 |
| 79 | MB-1 | Plaque (caries +) | EU620852 | EU620968 | EU621084 | EU621200 | EU603222 | EU621316 |
| 80 | T12-1 | Plaque (caries –) | EU620853 | EU620969 | EU621085 | EU621201 | EU603223 | EU621317 |
| 81 | T18-1 | Plaque (caries –) | EU620854 | EU620970 | EU621086 | EU621202 | EU603224 | EU621318 |
| 82 | MMRC12-1 | Soft lesion | EU620855 | EU620971 | EU621087 | EU621203 | EU603225 | EU621319 |
| 83 | R6630† | Knee fluid | EU620856 | EU620972 | EU621088 | EU621204 | EU603226 | EU621320 |
| 84 | R8152† | IUCD | EU620857 | EU620973 | EU621089 | EU621205 | EU603227 | EU621321 |
| 85 | R9841† | Aortic valve | EU620858 | EU620974 | EU621090 | EU621206 | EU603228 | EU621322 |
| 86 | R11372† | IUCD | EU620859 | EU620975 | EU621091 | EU621207 | EU603229 | EU621323 |
| 87 | R13240† | Subphrenic abscess | EU620860 | EU620976 | EU621092 | EU621208 | EU603230 | EU621324 |

| Study no. | Isolate | Origin | GenBank accession no. | | | | | |
|---|---|---|---|---|---|---|---|---|
| | | | *atpA* | *gltA* | *gyrA* | *metG* | *pgi* | *rpoB* |
| 88 | R14746† | Axilla | EU620861 | EU620977 | EU621093 | EU621209 | EU603231 | EU621325 |
| 89 | R19039† | Liver abscess | EU620862 | EU620978 | EU621094 | EU621210 | EU603232 | EU621326 |
| 90 | R21091† | Cerebral abscess | EU620863 | EU620979 | EU621095 | EU621211 | EU603233 | EU621327 |
| 91 | R23087† | Blood culture | EU620864 | EU620980 | EU621096 | EU621212 | EU603234 | EU621328 |
| 92 | R23275† | Blood culture | EU620865 | EU620981 | EU621097 | EU621213 | EU603235 | EU621329 |
| 93 | R23277† | Blood culture | EU620866 | EU620982 | EU621098 | EU621214 | EU603236 | EU621330 |
| 94 | R24330† | IUCD | EU620867 | EU620983 | EU621099 | EU621215 | EU603237 | EU621331 |
| **Reference and type strains** | | | | | | | | |
| 95 | P2G | | EU620868 | EU620984 | EU621100 | EU621216 | EU603238 | EU621332 |
| 96 | P6N | | EU620869 | EU620985 | EU621101 | EU621217 | EU603239 | EU621333 |
| 97 | P5K | | EU620870 | EU620986 | EU621102 | EU621218 | EU603240 | EU621334 |
| 98 | P6K | | EU620871 | EU620987 | EU621103 | EU621219 | EU603241 | EU621335 |
| 99 | P7K | | EU620872 | EU620988 | EU621104 | EU621220 | EU603242 | EU621336 |
| 100 | P8K | | EU620873 | EU620989 | EU621105 | EU621221 | EU603243 | EU621337 |
| 101 | P9K | | EU620874 | EU620990 | EU621106 | EU621222 | EU603244 | EU621338 |
| 102 | Pn4D | | EU620875 | EU620991 | EU621107 | EU621223 | EU603245 | EU621339 |
| 103 | Pn5D | | EU620876 | EU620992 | EU621108 | EU621224 | EU603246 | EU621340 |
| 104 | Pn1GA | | EU620877 | EU620993 | EU621109 | EU621225 | EU603247 | EU621341 |
| 105 | Pn16E | | EU620878 | EU620994 | EU621110 | EU621226 | EU603248 | EU621342 |
| 106 | P10N | | EU620879 | EU620995 | EU621111 | EU621227 | EU603249 | EU621343 |
| 107 | Pn6N | | EU620880 | EU620996 | EU621112 | EU621228 | EU603250 | EU621344 |
| 108 | Pn20E | | EU620881 | EU620997 | EU621113 | EU621229 | EU603251 | EU621345 |
| **109** | ***A. naeslundii* ATCC 12104$^T$** | | **EU620882** | **EU620998** | **EU621114** | **EU621230** | **EU603252** | **EU621346** |
| 110 | P5N | | EU620883 | EU620999 | EU621115 | EU621231 | EU603253 | EU621347 |
| 111 | CCUG 33920 | | EU620884 | EU621000 | EU621116 | EU621232 | EU603254 | EU621348 |
| 112 | CCUG 34725 | | EU620888 | EU621004 | EU621120 | EU621236 | EU603258 | EU621352 |
| 113 | CCUG 35334 | | EU620889 | EU621005 | EU621121 | EU621237 | EU603259 | EU621353 |
| 114 | CCUG 37599 | | EU620890 | EU621006 | EU621122 | EU621238 | EU603260 | EU621354 |
| 115 | CCUG 34285 | | EU620886 | EU621002 | EU621118 | EU621234 | EU603256 | EU621350 |
| 116 | CCUG 34286 | | EU620887 | EU621003 | EU621119 | EU621235 | EU603257 | EU621351 |
| 117 | CCUG 33932 | | EU620885 | EU621001 | EU621117 | EU621233 | EU603255 | EU621349 |
| **118** | ***A. johnsonii* CCUG 34287$^T$** | | **EU620891** | **EU621007** | **EU621123** | **EU621239** | **EU603261** | **EU621355** |
| **119** | ***A. oris* ATCC 27044$^T$** | | **EU620892** | **EU621008** | **EU621124** | **EU621240** | **EU603262** | **EU621356** |
| 120 | *A. viscosus* NCTC 10951$^T$ | | EU620893 | EU621009 | EU621125 | EU621241 | EU603263 | EU621357 |

*Caries +, Isolate from subject with active occlusal carie; caries –, isolate from caries-free subject.
†Isolates purchased from Dr Val Hall (Anaerobe Reference Unit, Department of Medical Microbiology and Public Health Laboratory, University Hospital of Wales, Cardiff, UK).

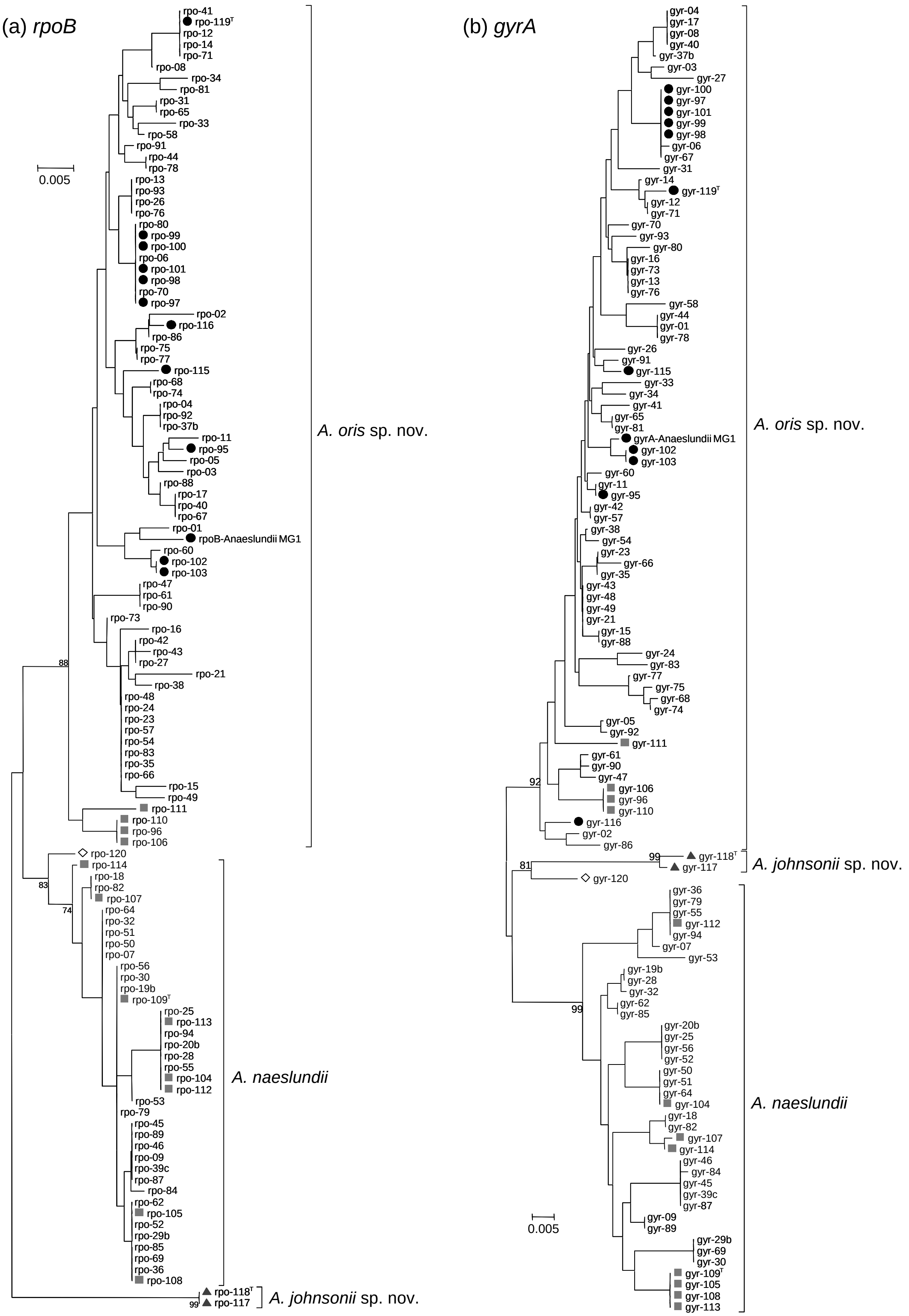


**Supplementary Fig. S1.** Phylogenetic relationships between *A. naeslundii* genospecies 1 (*A. naeslundii sensu stricto*), genospecies 2 (*A. oris* sp. nov.) and serotype WVA 963 (*A. johnsonii* sp. nov.). Neighbour-joining trees were determined by partial gene sequence analysis of *rpoB* (a), *gyrA* (b), *pgi* (c) and *gltA* (d). Type and reference strains are identified by study numbers (see Table 1) and indicated as follows: ●, *A. oris* sp. nov. (genosp. 2); ■, *A. naeslundii* (genosp. 1); ◇, *A. viscosus*; ▲, *A. johnsonii* sp. nov. (genosp. WVA 963). Bootstrap values are indicated at corresponding nodes. Bars, 0.005 substitutions per site.

**Henssge, U., Do, T., Radford, D. R., Gilbert, S. C., Clark, D. & Beighton, D. (2009).** Emended description of *Actinomyces naeslundii* and descriptions of *Actinomyces oris* sp. nov. and *Actinomyces johnsonii* sp. nov., previously identified as *Actinomyces naeslundii* genospecies 1, 2 and WVA 963. *Int J Syst Evol Microbiol* **59**, 509–516.

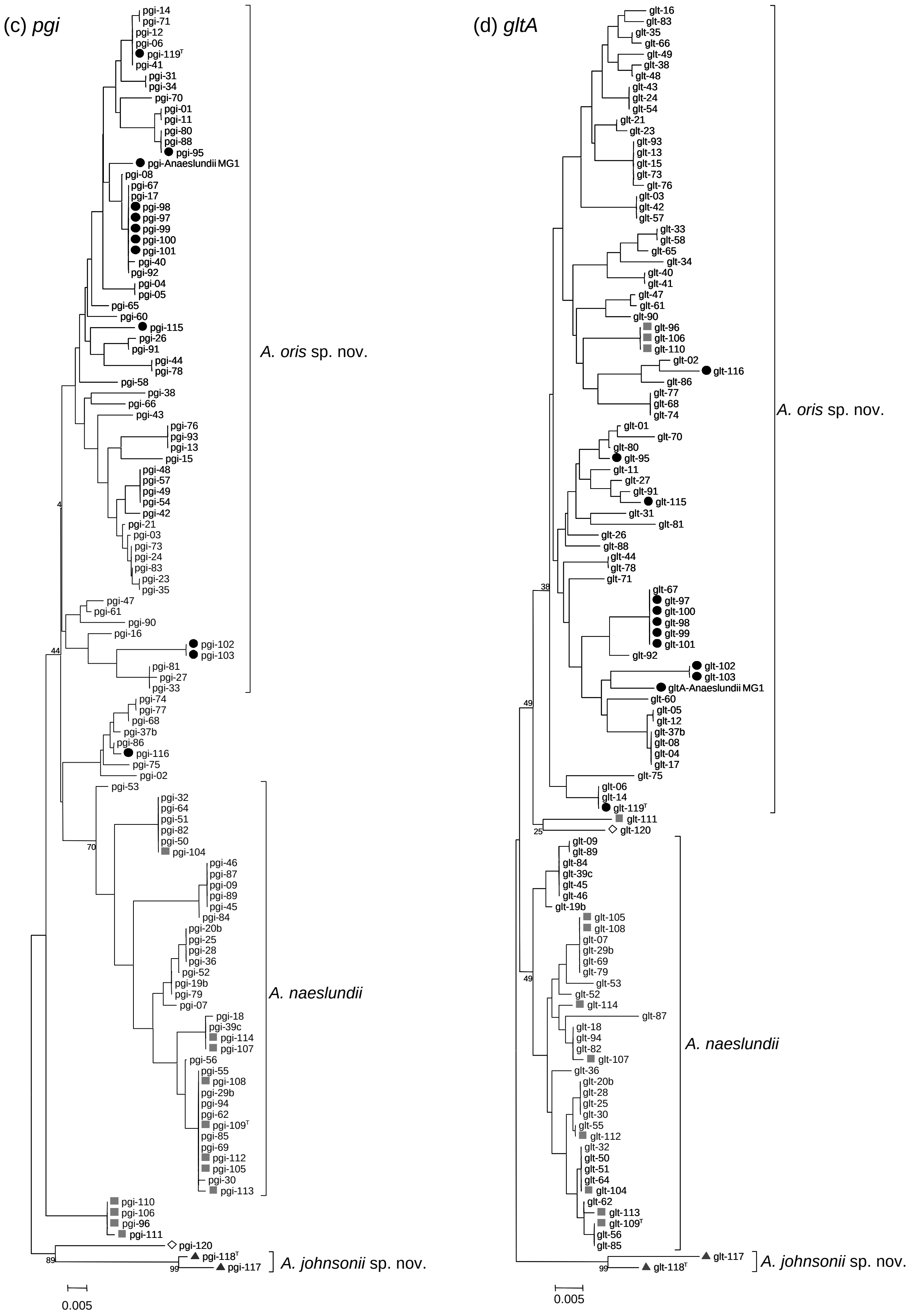


**Supplementary Fig. S1.** cont.
